# Supplementary figures and images for: Population structure and genetic connectivity of the scalloped hammerhead shark (Sphyrna lewini) across nursery grounds from the Eastern Tropical Pacific: Implications for management and conservation
Source: PLoS One. 2022 Dec 16;17(12):e0264879. doi: 10.1371/journal.pone.0264879 (PMC9757582; doi:10.1371/journal.pone.0264879)

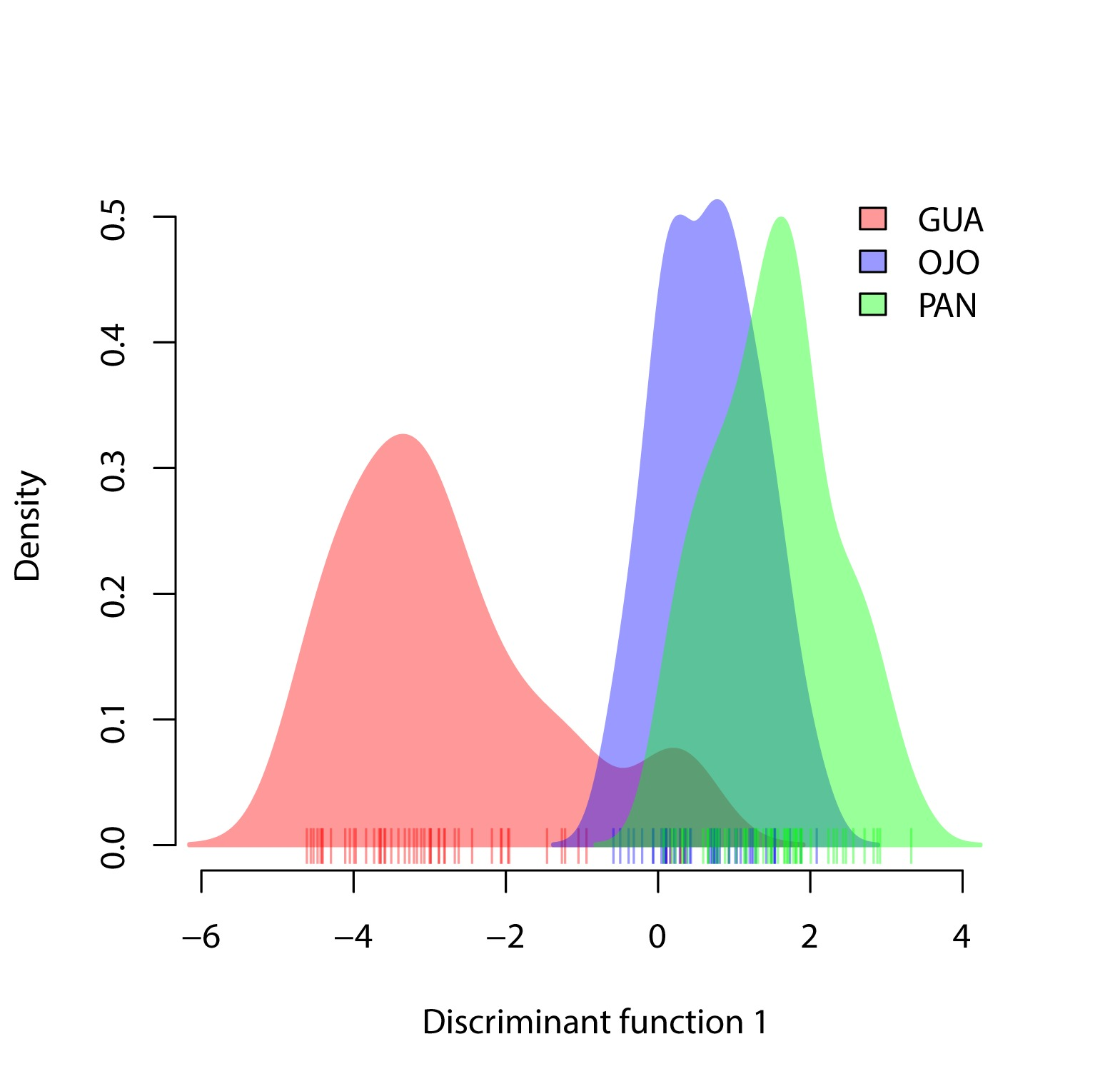

Supplement: S1 Fig — (TIF) [file pone.0264879.s001.tif]

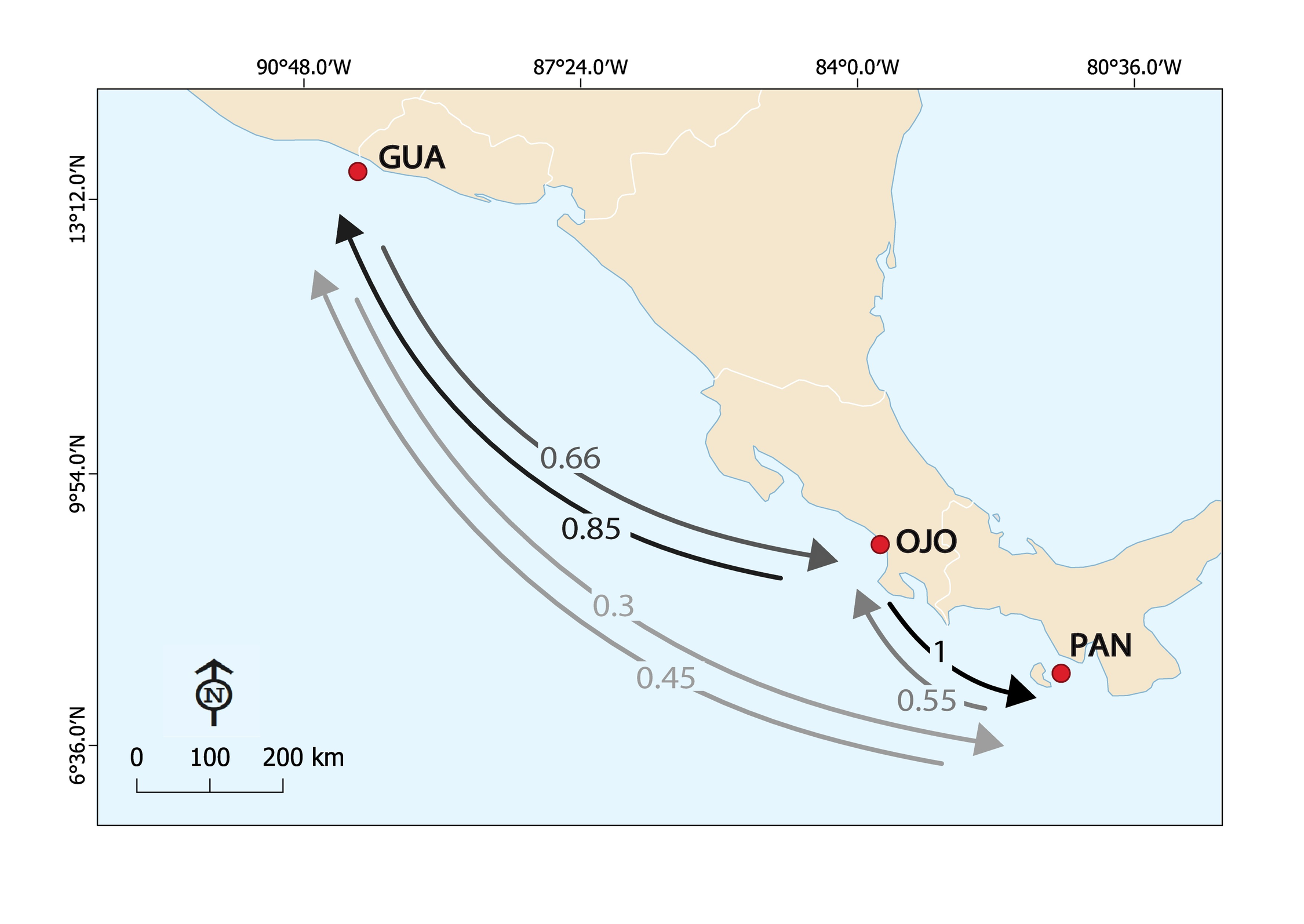

Supplement: S2 Fig — Arrows represent the relative number of migrants and estimated direction of gene flow between Guatemala (GUA), Costa Rica (OJO), and Panama (PAN). The darker the arrow, the higher the relative number of migrants between sampling locations. (TIF) [file pone.0264879.s002.tif]

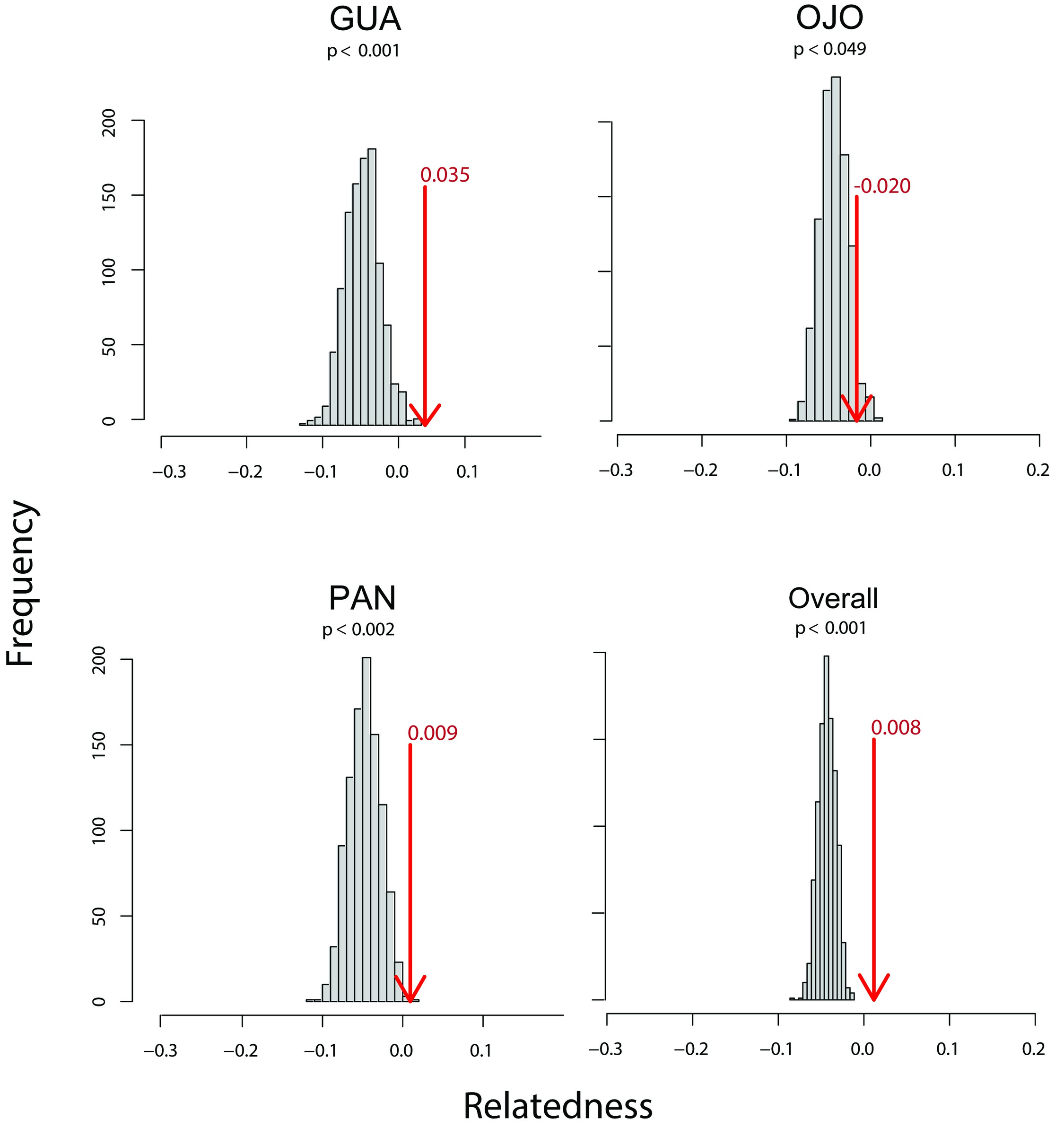

Supplement: S3 Fig — Expected distribution of average relatedness based on the Wang estimator of Sphyrna lewini in each sampling site and overall sampling sites using 1000 iterations. The average relatedness observed within sampling site and overall sampling site is the statistic test (observed in a red arrow). The further away the statistic test is from the simulated bars, the greater the significance of the relatedness test. (TIF) [file pone.0264879.s003.tif]

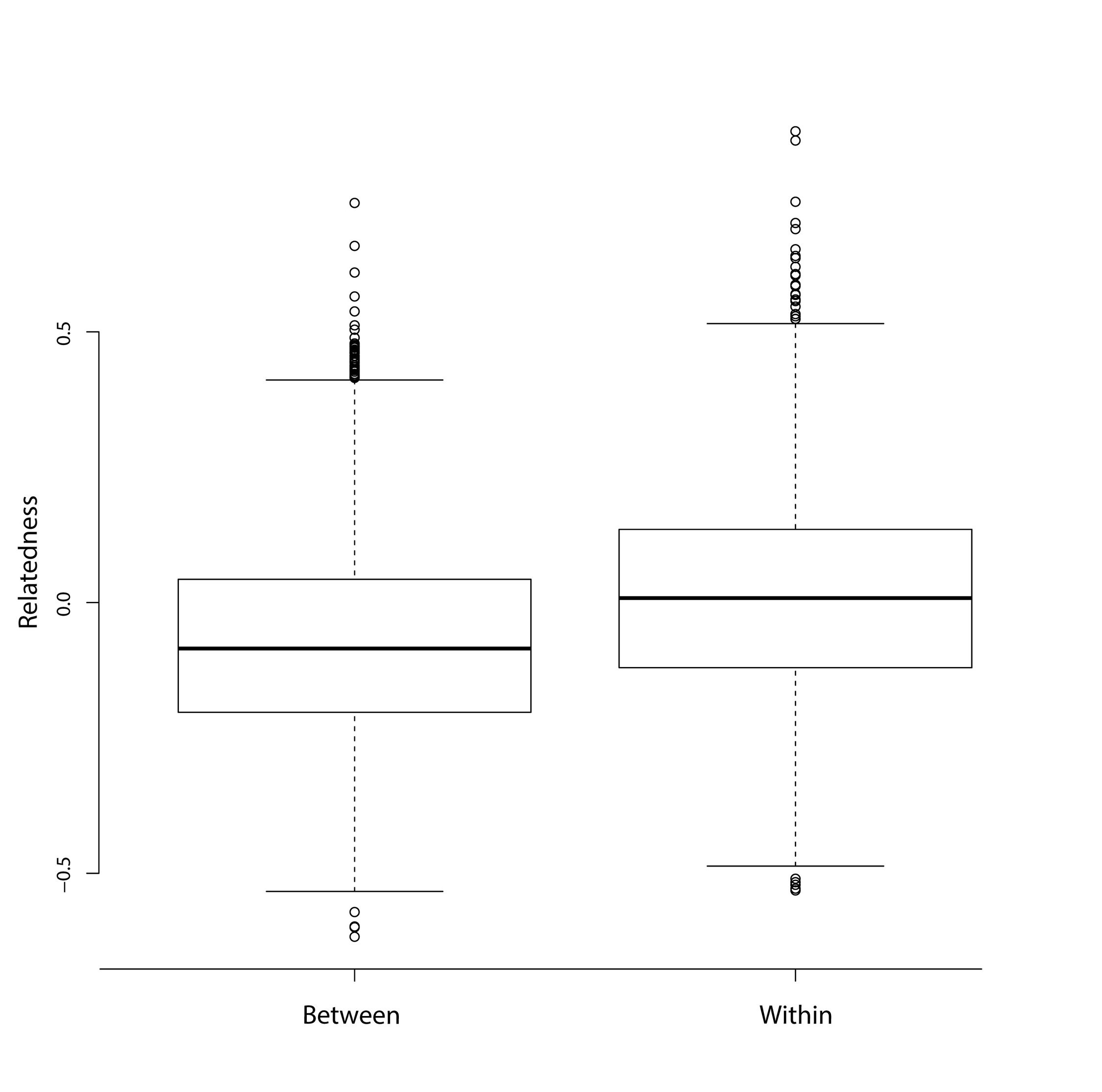

Supplement: S4 Fig — (TIF) [file pone.0264879.s004.tif]

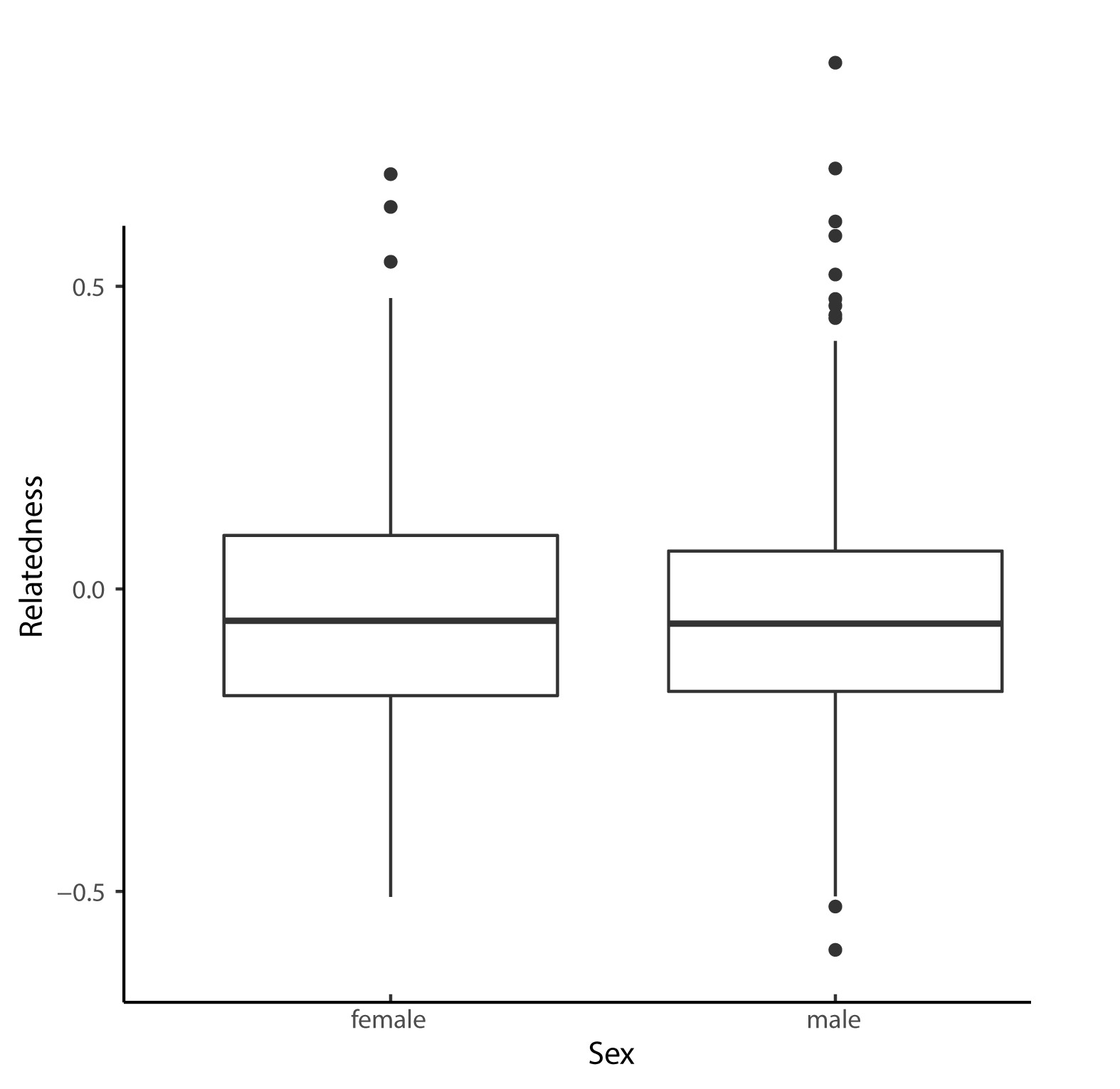

Supplement: S5 Fig — (TIF) [file pone.0264879.s005.tif]
