# Supplementary material for: Population structure and genetic connectivity of the scalloped hammerhead shark (Sphyrna lewini) across nursery grounds from the Eastern Tropical Pacific: Implications for management and conservation
Source: PLoS One. 2022 Dec 16;17(12):e0264879. doi: 10.1371/journal.pone.0264879 (PMC9757582; doi:10.1371/journal.pone.0264879)
Supplement: S1 Table — (PDF) [file pone.0264879.s006.pdf]

Table S1. Localities, the total number (n) and accession number of mtCR gene sequences for *Sphyrna lewini* from the Eastern Tropical Pacific.

Locality	N	Accession number	Author
Northern ETP			
**Mexico**			
**Baja California**	25	JN543266	Castillo-Olguin et al., 2012
**Sinaloa**	27	JN543266	Castillo-Olguin et al., 2012
	3	JN543267	Castillo-Olguin et al., 2012
	3	JN543268	Castillo-Olguin et al., 2012
	3	JN543270	Castillo-Olguin et al., 2012
**Nayarit**	19	JN543266	Castillo-Olguin et al., 2012
	1	JN543267	Castillo-Olguin et al., 2012
	1	JN543268	Castillo-Olguin et al., 2012
	4	JN543269	Castillo-Olguin et al., 2012
**Michoacán**	13	JN543266	Castillo-Olguin et al., 2012
	3	JN543267	Castillo-Olguin et al., 2012
	1	JN543268	Castillo-Olguin et al., 2012
**Oaxaca**	8	JN543266	Castillo-Olguin et al., 2012
**Chiapas**	14	JN543266	Castillo-Olguin et al., 2012
Central-southern ETP			
**Guatemala**			
Las Lisas	72	OL692109 - OL692180	(This study)
**Costa Rica**			
Ojochal	43	OL692181 - OL692223	(This study)
Coyote	34	OL692224 - OL692257	(This study)
Isla del Coco	15	OL692258 - OL692272	(This study)
**Panama**			
Punta Chame	65	OL692273 - OL692337	(This study)
**Colombia**			
Malpelo Island	8	DQ438152.1	Duncan et al., 2006
	9	DQ438153.1	Duncan et al., 2006
	1	DQ438159.1	Duncan et al., 2006
Port Buenaventura	5	DQ438152.1	Duncan et al., 2006
	14	DQ438153.1	Duncan et al., 2006
	1	DQ438159.1	Duncan et al., 2006
	1	GUO14391	Chapman et al., 2009
Sanquianga	6	DQ438152.1	Duncan et al., 2006
	13	DQ438153.1	Duncan et al., 2006
	1	GUO14391	Chapman et al., 2009
Utria	9	DQ438152.1	Duncan et al., 2006
	12	DQ438153.1	Duncan et al., 2006
