# Supplementary material for: Population structure and genetic connectivity of the scalloped hammerhead shark (Sphyrna lewini) across nursery grounds from the Eastern Tropical Pacific: Implications for management and conservation
Source: PLoS One. 2022 Dec 16;17(12):e0264879. doi: 10.1371/journal.pone.0264879 (PMC9757582; doi:10.1371/journal.pone.0264879)
Supplement: S2 Table — Ta: Annealing temperature, Ho: Observed heterozygosity, He: Expected heterozygosity, Ar: Allelic richness, Na: Number of alleles, Ua: Unique alleles, Fis: Inbreeding coefficient. (PDF) [file pone.0264879.s007.pdf]

Table S2. Genetic diversity indexes of each microsatellite loci from *Sphyrna lewini* individuals in the Eastern Tropical Pacific. Ta: annealing temperature, Ho: observed heterozygosity, He: expected heterozygosity, Ar: allelic richness, Na: number of alleles, Ua: unique alleles, Fis: inbreeding coefficient.

**Locus**	**Ta**	**Ho**	**He**	**Ar**	**Na**	**Ua**	**Fis**
Sle013	60	0.607	0.639	3.901	10	8	0.0668
Sle038	59	0.795	0.863	8.589	15	4	0.0595
Sle045	60	0.673	0.663	4.414	5	0	0.0031
Sle054	59	0.5394	0.718	5.072	11	3	0.0146
Sle071	60	0.562	0.61	5.603	14	5	0.0854
Sle081	57	0.778	0.827	7.552	11	1	0.0144
Sle086	60	0.680	0.746	4.960	8	2	0.0465
Sle089	60	0.836	0.884	10.169	19	4	0.0269
Sle033	59	0.733	0.795	7.654	9	2	0.0745
