# Supplementary material for: Population structure and genetic connectivity of the scalloped hammerhead shark (Sphyrna lewini) across nursery grounds from the Eastern Tropical Pacific: Implications for management and conservation
Source: PLoS One. 2022 Dec 16;17(12):e0264879. doi: 10.1371/journal.pone.0264879 (PMC9757582; doi:10.1371/journal.pone.0264879)
Supplement: S3 Table — (PDF) [file pone.0264879.s008.pdf]

Table S3. Geographic distribution and frequency of mitochondrial control region haplotypes of *Sphyrna lewini* individuals from the Eastern Tropical Pacific.

		GUA	OJO	COY	ICO	PAN	NAY	OAX	MCH	BJC	CHP	SIN	PTB	UTR	SNQ	MLP
N		(72)	(44)	(34)	(15)	(65)	(30)	(9)	(20)	(30)	(16)	(41)	(21)	(21)	(20)	(18)
	Accession #															
Hap1	OL692109	2	0	0	0	1	0	0	0	0	0	0	0	0	0	0
Hap2	OL692111	1	0	0	0	0	0	0	0	0	0	0	0	0	0	0
Hap3	OL692112	2	0	0	0	0	0	0	0	0	0	0	0	0	0	0
Hap4	OL692114	39	29	20	10	36	7	0	4	0	0	3	14	12	13	9
Hap5	OL692151	23	11	13	4	23	23	9	16	30	16	34	5	9	6	8
Hap6	OL692174	3	0	0	0	0	0	0	0	0	0	0	0	0	0	0
Hap7	OL692177	2	0	0	0	0	0	0	0	0	0	0	0	0	0	0
Hap8	OL692181	0	1	0	0	2	0	0	0	0	0	0	1	0	0	1
Hap9	OL692211	0	1	0	0	0	0	0	0	0	0	0	0	0	0	0
Hap10	OL692213	0	1	0	1	1	0	0	0	0	0	0	0	0	0	0
Hap11	OL692214	0	1	0	0	0	0	0	0	0	0	0	0	0	0	0
Hap12	OL692231	0	0	1	0	0	0	0	0	0	0	0	0	0	0	0
Hap13	OL692298	0	0	0	0	1	0	0	0	0	0	0	0	0	0	0
Hap14	OL692324	0	0	0	0	1	0	0	0	0	0	0	0	0	0	0
Hap15	JN543270	0	0	0	0	0	0	0	0	0	0	3	0	0	0	0
Hap16	GU014391	0	0	0	0	0	0	0	0	0	0	0	1	0	1	0
