# Supplementary material for: Population structure and genetic connectivity of the scalloped hammerhead shark (Sphyrna lewini) across nursery grounds from the Eastern Tropical Pacific: Implications for management and conservation
Source: PLoS One. 2022 Dec 16;17(12):e0264879. doi: 10.1371/journal.pone.0264879 (PMC9757582; doi:10.1371/journal.pone.0264879)
Supplement: S4 Table — Significant values α = 0.05, are presented in bold. (PDF) [file pone.0264879.s009.pdf]

Table S4. Pairwise fixation indexes (D_{EST} and F_{ST}) with lower and upper 95% confidence intervals (CI), between sampling areas of the Eastern Tropical Pacific. Significant values α = 0.05, are presented in bold.

	mean	Lower 95% CI	Upper 95%CI
**D_{EST}**			
GUA vs. OJO	**0.0254**	0.0046	0.0554
GUA vs. PAN	**0.0734**	0.0434	0.1096
GUA vs. ICO	**0.0415**	0.0102	0.0895
OJO vs. PAN	**0.029**	0.0093	0.0531
OJO vs. ICO	0.007	-0.0181	0.0466
PAN vs. ICO	0.003	-0.0206	0.0431
**F_{EST}**			
GUA vs. OJO	**0.0587**	0.0417	0.0772
GUA vs. PAN	**0.0807**	0.0624	0.1003
GUA vs. ICO	**0.0801**	0.0584	0.1047
OJO vs. PAN	**0.0185**	0.0089	0.0296
OJO vs. ICO	0.0093	-0.0053	0.0292
PAN vs. ICO	0.0064	-0.0093	0.0283
