# Supplementary material for: Population structure and genetic connectivity of the scalloped hammerhead shark (Sphyrna lewini) across nursery grounds from the Eastern Tropical Pacific: Implications for management and conservation
Source: PLoS One. 2022 Dec 16;17(12):e0264879. doi: 10.1371/journal.pone.0264879 (PMC9757582; doi:10.1371/journal.pone.0264879)
Supplement: Attachment — Submitted filename: Response to Reviewers.docx [file pone.0264879.s012.docx]

May 20^th^, 2022

San José, Costa Rica

To Dr. Johann Mourier, Academic Editor;

Dr. Pierre Feutry, Referee;

and two anonymous referees:

First of all, thank you so much for your time to revise the manuscript. Your comments have been extremely helpful to improve this manuscript. Here below I address all of your comments. First I will start with observations about Journal Requirements and then I will address the comments regarding the content of the manuscript that were provided by each Referee.

I look forward to you receiving the revised manuscript and expect that it now can meet PLOS ONE’s publication criteria.

Kind regards,

Mariana

A. **Regarding the comments about Journal Requirements:**
1. Please ensure that your manuscript meets PLOS ONE's style requirements, including those for file naming.

Yes thank you, I double checked that the manuscript has PLOS ONE’s style requirements.

2. We note that you have provided funding information. However, funding information should not appear in the Funding section or other areas of your manuscript.

Thank you, yes I eliminated the funding statement from the manuscript.

3. We note that you have stated that you will provide repository information for your data at acceptance.

The data of mitochondrial control region sequences was deposited in Genbank and will not be released to the public database until December 2033,or until the data or accession numbers appear in print, whichever
is first.

4. We note that Figure 1 in your submission contain map image which may be copyrighted

I specified in the methods section that the map images were created by a public domain without any copyright.

B. **Regarding the comments about the content of the Manuscript:**

**Reviewer #1:**

Thank you again Dr. Pierre Feutry, your comments were very welcome. Here I respond to your observations:

1. L87-91: there are other philopatric shark species, look up genus Glyphis and Bull Shark

Thank you, I added these species to the introduction.

2. L112: are the acronyms ok for the locations in Costa Rica? There is a discrepancy between that sentence (COY, OJO and COS) and figure 1 (COY, OJO, ICO). Cocos Island seems to be missing in the locations from CR

Yes, I missed mentioning Cocos Island in this section. For microsatellite analyses I only use the sampling site Ojochal of Costa Rica, that is why I was using the acronym OJO and COS for the same area, I understand this is confusing. I will change it.

3. L123-124: along the coast is quite vague and doesn’t really match up with the specificity of the sampling locations.

Noted! And changed.

4. L149: “Nance et al. (2009 lewini” Typos?

Yes! I did not see it before

5. L167 : add a « , » after « among haplotypes”

Yes, much better

6. L191-192: related individuals should only be removed if there is a reason to think there was a bias in the sampling favoring the catch of related individuals, e.g. 2 neonates caught in the same eddy on the same day. On the opposite, if two siblings were caught 3 years apart there is no reason to remove one of them. See paper by Anderson & Waples (2017) for further details

I ran all analyses with both data sets and the difference was very little, the total number of individuals that I removed from the analyses were 19, the majority were from Guatemala. No strong conclusion was different. Something that could further justify not removing these pairs of Full Siblings, is that most of them were sampled one year apart, and some even in different countries, so I would not trust that they are really full siblings. I checked the distribution of my data comparing it to what is expected of Unrelated pairs, Half Siblings, Full Sibling and Parent Offspring pairs (Fig 3). The majority are distributed as Unrelated, so I decided to leave all analyses with the full data set.

7. L213-216: need to explain what method was used to choose the “best” K

Yes, the Evanno method was used

8. L220-221: how did you deal with the potential of overfitting? Alpha score? Cross-validation?

In the DAPC, retaining too many PCA axes with respect to the number of individuals can lead to over-fitting in the membership probabilities. To decide in an objective way how many PCA axes to retain, a cross validation analysis was performed with the xvalDapc function from the Adegenet package in R. This function tries different numbers of axes and the quality of the corresponding DAPC is assessed by cross-validation. The number of PCA axes associated with the lowest Mean Squared Error (40 PCs) were then retained in the DAPC.

Jombart T, Devillard S and Balloux F (2010) Discriminant analysis of principal components: a new method for the analysis of genetically structured populations. BMC Genetics11:94. doi:10.1186/1471-2156-11-94

9. L238: delete “relatively low”, this type of comment on the results belongs to the discussion.

You are right, I will delete it

10. L273: What is significant? Less than 0.05 p-value after correction? It’s generally better to provide the confidence interval for the Fst values rather than p-values, could add them in the upper diagonal

The significance level was 0.05, so yes less tan 0.05 and there was no correction applied. For mitochondrial DNA Fst pairwise comparisons I could not find a way to provide the confidence intervals. I added the p-values to the upper diagonal.

11. L284: delete “duly”

Deleted

12. L305-306: move “(Fis)” after “inbreeding coefficients”

Yes!

13. L309-311: replace “a greater” and “a lower” by “the greatest” and “the lowest” respectively

Thank you yes much better

14. L312: replace “less” by “the least” and “greater” by “the most”

Yes

15. L368: most likely a bottleneck is not detected because the population size is not (yet) low enough, is that what you mean by “only a few generations have passed since overfishing started”. Please reword

After consideration, I think that these results of the bottleneck analysis are not relevant. Other reviewers also suggested to eliminate this since it does not contribute much.

16. L368-369: how can you report a “non-detectable bottleneck”? Do you mean that no bottleneck was detected?

After consideration, I think that these results of the bottleneck analysis are not relevant. Other reviewers also suggested to eliminate this since it does not contribute much.

17. L379: the entire mitogenome is one marker, I would reword and say “using more mitochondrial regions”

Yes, this makes more sense

18. L380: Feutry et al. 2014 is a better reference for that statement

Yes!

19. L404: or more markers, or both. See Foster et al. 2021

Yes, I understand what you mean, it is important to use robust sample sizes or more markers or both. The thing is that here, I want to emphasize in that what out study has that is not seen in Nance et al., 2009 is a bigger sample size.

20. L415: not a challenge if you have plenty of markers (generally SNPs)

Ok, I will specify genotypic data of few loci.

21. L430: quite likely there is further structuring, but more markers and/or more individuals are required to demonstrate it. I think the discussion need to emphasize a bit more the high potential for finer scale structure and the need to look into it with appropriate genetic techniques

Yes, I added this to the discussion

**Reviewer #2**

Thank you again for all your constructive comments, here I address each of them. And made the corresponding improvements to the manuscript.

1. The data for HWE are not presented, so the p-values are unknown, as is the scale for these analyses. Was HWE significant calculated for all individuals pooled or for each sample site? This is important because of the assumptions of HWE (e.g. pooling two populations or not-pooling a single population could cause violations in the HWE assumptions).

The data for HWE was calculated for all individuals pooled and for each sampling site separately, these results are presented in the Microsatellite loci section. There were no significant deviations from HWE after Holm-Bonferroni correction of the 9 loci that were used for analyses.

2. Line 64-65: All populations experience genetic drift, but the effects are more pronounced in small populations. Suggest rephrasing to make this clear.

You are right I rephrased it the following way:

Population level declines are of major concern in conservation since the effects of genetic drift and inbreeding are pronounced in small populations, which may lead to loss of genetic diversity and compromise the ability of a population to cope with environmental change.

3. Line 98: Suggest rephrasing from “sorting out the genetic diversity…”. For example, could change to “it is important to assess population structure and genetic diversity between potential nursery areas in this region”.

Perfect, I rephrased this the following way:

Given the limited data on the population structure of *S. lewini* and the high fishing pressure that this species is currently under throughout the ETP, it is important to assess sorting to assess population structure and genetic diversity in potential nursery areas of the region, to develop effective management and conservation strategies.

4. Line 100-103: Remove, as this is methods.

Yes, I removed those lines.

5. Line 112: Add sample sizes for each site.

Yes, I could do this but it would be a little confusing, since the samples that were used for the mitochondrial control region are not exactly the same as the ones used for the microsatellite loci analyses. I am going to add the total number of samples I had from each location

6. Line 123: Here is says samples were collect from juveniles, but the introduction said YOY. Which is it? Similarly, what life stages were sampled in the other studies where the data is used here? This needs to be stated, and potential caveats addressed in the discussion since life stage sampled is needed to interpret the data more fully.

The Castillo-Olguin and collaborators (2009) study sampled juveniles (60-130cm long).Nance and collaborators (2011) paper were juveniles 1-3 years old except adults in Manta Island (TL ≥ 1.5m). Quintanilla and collaborators (2015) juveniles 30-50 TL except Malpelo Island (adults TL ≥ 1.5m). Thank you I cleared this in methods

7. Line 153: The entire mtDNA control region?

Yes, this was modified

8. Line 136: Remove “species-specific”. These primers may have been designed for S. lewini, but that does not mean they are species-specific. That would involve cross testing in other species to make sure they do not cross-amplify DNA from other species.

Yes! You are right. Removed

9. Lines 146, 159: More details are needed for how PCR products were cleaned and sequenced, as well as for fragment analysis, e.g. what size standard was used?

Yes, for microsatellites I sequenced all loci in both directions to verify the microsatellite dinucleotide motifs. Then for fragment analyses it was done with a 5-dye chemistry (FAM, NED, PET, VIC) and an internal size standard LIZ GS500

10. Line 156: Suggest including cycle numbers in Table S2 since they were not the same across all loci.

Yes, this was from the initial protocol (8-20 cycles) I only used 8 cycles

11. Line 165: Add sample sizes for these locations.

Ready

12. Line 173: Suggest exact tests here too.

Yes, as you suggested I ran the exact test of population differentiation, in Arlequin. The results are almost exactly the same as the Fst values. I included the significance of the exact test in Table 2.

These are the Fst Values:

|  | BJC | SIN | NAY | MCH | OAX | CHP | GUA | COY | OJO | PAN | ICO | UTR | PTB | SNQ | MLP |
| --- | --- | --- | --- | --- | --- | --- | --- | --- | --- | --- | --- | --- | --- | --- | --- |
| BJC | - |  |  |  |  |  |  |  |  |  |  |  |  |  |  |
| SIN | 0.049 | - |  |  |  |  |  |  |  |  |  |  |  |  |  |
| NAY | **0.208** | 0.027 | - |  |  |  |  |  |  |  |  |  |  |  |  |
| MCH | 0.169 | 0.009 | -0.039 | - |  |  |  |  |  |  |  |  |  |  |  |
| OAX | 0.000 | -0.015 | 0.101 | 0.050 | - |  |  |  |  |  |  |  |  |  |  |
| CHP | 0.000 | 0.020 | 0.151 | 0.105 | 0.000 | - |  |  |  |  |  |  |  |  |  |
| GUA | **0.395** | **0.175** | **0.163** | **0.205** | **0.329** | **0.359** | - |  |  |  |  |  |  |  |  |
| COY | **0.510** | **0.134** | **0.179** | **0.239** | **0.403** | **0.449** | -0.009 | - |  |  |  |  |  |  |  |
| OJO | **0.549** | **0.194** | **0.270** | **0.323** | **0.461** | **0.499** | 0.007 | 0.001 | - |  |  |  |  |  |  |
| PAN | **0.406** | **0.163** | **0.158** | **0.203** | **0.335** | **0.367** | -0.002 | -0.017 | -0.003 | - |  |  |  |  |  |
| ICO | **0.661** | **0.123** | **0.258** | **0.325** | **0.502** | **0.575** | -0.020 | -0.029 | -0.037 | -0.031 | - |  |  |  |  |
| UTR | **0.575** | **0.103** | **0.170** | **0.240** | **0.435** | **0.497** | -0.018 | -0.038 | -0.003 | -0.027 | -0.034 | - |  |  |  |
| PTB | **0.366** | **0.106** | **0.169** | **0.181** | **0.231** | **0.291** | 0.026 | 0.014 | -0.001 | 0.019 | -0.025 | 0.003 | - |  |  |
| SNQ | **0.352** | **0.090** | **0.153** | **0.165** | **0.211** | **0.273** | 0.022 | 0.009 | 0.002 | 0.016 | -0.027 | -0.004 | -0.049 | - |  |
| MLP | **0.532** | **0.086** | **0.141** | **0.201** | **0.377** | **0.446** | -0.016 | -0.035 | -0.005 | -0.030 | -0.030 | -0.048 | -0.005 | -0.008 | - |
|  |  |  |  |  |  |  |  |  |  |  |  |  |  |  |  |

These are the exact tests of population differentiation α= 0.05:

|  | BJC | SIN | NAY | MCH | OAX | CHP | GUA | COY | OJO | PAN | ICO | UTR | PTB | SNQ | MLP |
| --- | --- | --- | --- | --- | --- | --- | --- | --- | --- | --- | --- | --- | --- | --- | --- |
| BJC | - |  |  |  |  |  |  |  |  |  |  |  |  |  |  |
| SIN | - | - |  |  |  |  |  |  |  |  |  |  |  |  |  |
| NAY | **+** | - | - |  |  |  |  |  |  |  |  |  |  |  |  |
| MCH | - | - | - | - |  |  |  |  |  |  |  |  |  |  |  |
| OAX | - | - | - | - | - |  |  |  |  |  |  |  |  |  |  |
| CHP | - | - | - | - | - | - |  |  |  |  |  |  |  |  |  |
| GUA | **+** | **+** | **+** | **+** | + | **+** | - |  |  |  |  |  |  |  |  |
| COY | **+** | **+** | **+** | **+** | **+** | **+** | - | - |  |  |  |  |  |  |  |
| OJO | **+** | **+** | **+** | **+** | **+** | **+** | - | - | - |  |  |  |  |  |  |
| PAN | **+** | **+** | **+** | **+** | **+** | **+** | - | - | - | - |  |  |  |  |  |
| ICO | **+** | **+** | **+** | **+** | **+** | **+** | - | - | - | - | - |  |  |  |  |
| UTR | **+** | **+** | **+** | **+** | **+** | **+** | - | - | - | - | - | - |  |  |  |
| PTB | **+** | **+** | **+** | **+** | **+** | **+** | - | - | - | - | - | - | - |  |  |
| SNQ | **+** | **+** | **+** | **+** | **+** | **+** | - | **-** | - | - | - | - | - | - |  |
| MLP | **+** | **+** | **-** | **-** | **+** | **+** | - | - | - | - | - | - | - | - | - |
|  |  |  |  |  |  |  |  |  |  |  |  |  |  |  |  |

13. Line 191-192: I understand why the authors removes FS from the analyses, but does this then impact population structure and genetic diversity statistics in the other direction? Since relatedness/sibship approaches can be used to elucidate population structure and natal philopatry, it might be worth exploring the data without this removal of FS and/or going further with relatedness analyses. See next comment as well.

Yes, I ran all analyses with both sets of data and the difference is minimal, no relevant conclusions changed. I explored a little further with relatedness analyses.

Another reviewer made the observation that related individuals should only be removed if there is a reason to think there was a bias in the sampling favoring the catch of related individuals, e.g. 2 neonates caught in the same eddy on the same day. On the opposite, if two siblings were caught 3 years apart there is no reason to remove one of them. See paper by Anderson & Waples (2017) for further details.

Something that could further justify not removing these pairs of Full Siblings, is that most of them were sampled one year apart, and some even in different countries, so I would not trust that they are really full siblings.

14. Line 202-204: Were these comparisons also made between individuals at different sites? Suggest doing so to compare to the within-nursery data. It would also be interesting to look at sibship between different nursery areas as well as within nursery areas.

Yes, I added some analyses and graphs that explored further the relatedness within and between nursery areas.

15. Line 210: Why not calculate DEST for mtDNA too?

I could not find any software or R package that would calculate Dest values for mtDNA data.

16. Line 213: Suggest a hierarchical STRUCTURE if any of the identified populations (GUA, COS, PAN) have >1 sampling site. This is not really clear to me, as per the below comment for line 282. Also suggest looking at delta K

For this analyses there was only one sampling site per identified population. What I mean by this is only one sampling area in Guatemala, one in Costa Rica and one in Panamá. I also now present STRUCTURE plots with different values of K, and specify that the number of clusters identified by the Evanno method was K = 2.

17. Table 1: Suggest calculating genetic diversity indices for each of the identified populations; this will give an estimate of diversity at scales relevant to management.

Yes very good idea, thank you I added this.

18. Line 250-252: Suggest rephrasing these sentences as they are confusing. I suspect the authors are trying to say that there were two common haplotypes across all sampling sites, but they were found at different frequencies in Mexico compared to Central America and Colombia.

Yes, I cleared this

19. Line 262-265: This statement is long and confusing. An AMOVA should be set up to test a specific hypothesis.

Yes, here I added different configurations of the data for the AMOVA analyses. The two group configuration (Northern ETP and Central-southern ETP), is the one that best explains the variation found. I specified the AMOVA as a hypothesis in the following way:

In order to observe which configuration of the data best explained the variance, the AMOVA was performed with three different groupings: 1) one region (all locations); 2) two regions the Northern Eastern Tropical Pacific (Mexico) and the Central-southern Eastern Tropical Pacific (Guatemala, Costa Rica, Panama and Colombia); and 3) three regions the Northern Eastern Tropical Pacific (Mexico collection sites), the Central Eastern Pacific (Guatemala, Costa Rica and Panama), and the Southern Eastern Tropical Pacific (Colombia).

20. Line 282: I’m finding the acronyms and verbiage surrounding locations somewhat confusing. Here, GUA, COS, and PAN are mentioned. GUA and PAN are labelled on the map, but COS is not. I’m guessing that COS includes samples from >1 site, but the same may also be true for PAN based on the map. Later, there is reference to regions (e.g., 372) but it is difficult to follow given the inconsistencies. Suggest explaining sampling sites/ countries (pooled or not), regions (countries pooled?), etc. early on and then using the same language throughout the manuscript.

Yes, thank you I have cleared this misunderstanding. From now on I refer only to Ojochal (OJO), as it was the only location from Costa Rica that was analyzed with microsatellites.

21. Line 285: “population-specific”- what was this defined by?

Sorry yes, I meant sampling location.

22. Line 291: Were all FS pairs from the same sampling site? This could be an interesting discussion point.

From the FS pairs that I found with 99% confidence, 17 were from the same sampling site. From the 5 that were from different sampling sites: 2 pairs were found between Costa Rica and Guatemala, and three pairs between Costa Rica and Panamá. I decided to not go further into these results, since there were inconsistencies when changing the alpha value. ML related identified as much as 200 pairs of FS in the data. This does not make sense, since they were pairs from different sampling sites and also from different years. I decided to maintain the analyses presented in Fig 3, in which the distribution of my data, is similar to that of Unrelated pairs (using the same allele frequencies as my data).

23. Line 308, 311, etc: Suggest reporting actual P-value. Was a correction applied to these statistical tests? If so, what was the new threshold?

Dest values and Weir and & Cockerham’s Fst values were calculated with the function fastDivPart in the R package diversity. The variance of these statistics was assessed by 100000 bootstrap iterations. For pairwise calculations carried out by the function, a bias corrected 95% confidence interval is calculated. I calculated the 95% confidence interval for these statistics

24. Line 316-319: These statements seem to be contradictory. Were they all non-significant?

Yes, this was written in a confusing way. What this analysis did was test if there was asymmetric gene flow between these areas, which was not found. The statement should be corrected the following way:

Analysis of the extent and direction of gene flow showed no significant asymmetric movement between coastal sampling sites.

25. Line 321: Why were the samples from the Cocos Islands not included in analyses? For example, FST, DEST, Structure, etc.? The sample size wasn’t huge, but still worth including in the structure plot at a minimum.

Yes, I will ran these analyses with samples from Cocos included, and now present these results.

26. Line 339-340: The statement “The average withing sampling site….” is confusing; suggest rephrasing.

Thank you, yes. This should be: The average relatedness examined within sampling sites and overall sampling sites is the statistic test (observed in a red arrow).

27. Line 353 and elsewhere: Population declines can lead to a loss of genetic diversity, but that does not mean that: 1) population declines always cause declines in genetic diversity, or 2) that all populations with low diversity have undergone recent declines. This section seems to attribute the observed levels of genetic diversity to recent population declines, but this is not actually known. Elasmobranchs have some of the slowest rates of mutation among vertebrates, so genetic diversity accumulates slowly and can be low even in the absence of population declines. Suggest developing this section to be more comprehensive of genetic diversity in elasmobranchs, perhaps bringing in phylogeography (e.g. how recently might these populations been founded?)

Yes, I re-wrote this part of the Discussion.

28. Line 356: Levels of genetic diversity were not calculated for the central-southern ETP overall- they were calculated by sampling sites from what I can tell. Suggest analyzing genetic diversity for the identified populations to back up this statement. It also makes more sense from a management perspective to analyze data for each identified population.

Yes! I added these results.

29. Line 358-359: Take care with verbiage. Genetically distinct populations do not mean they resulted from independent evolutionary history. All populations of this species in the ETP likely have a common evolutionary history. This is evidenced by the presence of two common haplotypes shared across populations.

Yes, I re-wrote this part of the Discussion.

30. Line 367: It is stated that a bottleneck was not detected, but no data are presented to support this. Suggest either including bottleneck tests (with discussion on caveats of the various statistical approaches) or deleting the statement about the detection of bottleneck tests.

Yes, I consider deleting the statement is better. This is not a relevant result.

31. Line 373: The sentence on this line is confusing, suggest rephrasing.

Yes, I rephrased it the following way:

This pattern is mainly due to an uneven distribution of the two most common haplotypes, one is found in higher frequency in the Northern ETP while the other is found in higher frequency in the Central-southern ETP.

32. Line 376: The term “sub-population’ has a specific meaning for the IUCN species assessments, which is cited as the source of this definition. The IUCN definition of ‘sub-population’ is not the same as used in population genetics. I suggest the authors read this definition more carefully and rework this point. The data presented in this paper does not support further splitting the EP sub-population of this species, as per the IUCN definition. Within this region, the identification of distinct population units is important to inform management, so suggest focusing on that.

Thank you, yes I had not considered the strict definition of subpopulation of the IUCN, which specifies that they are groups with very little exchange. In this case I was referring specifically to a distinct population segments, defined under the Endangered Species Act as a vertebrate population or group of populations that is discrete from other populations of the species and significant in relation to the entire species.

33. Line 388-391; 397; 400-401; 436: The phrasing on these lines need some work as it is difficult to understand/follow. For example, line 388 mentions oceanographically dynamic regions and then uses the phrase ‘mixing zone’. Is this referring to a physical mixing zone or ‘mixing’ meaning gene flow?

Yes, in this study (Rodríguez-Zárate et al., 2018) make a simulation of what would happen if they freed a particle from a coastal area along the coast of Central America and Mexico, where would the particle drift depending on oceanographic conditions, so here they are referring to a physical mixing zone.

34. Line 400 mentions “high sampling effort” but not what locations fit this category. Etc.

Yes I rephrased this.

35. Line 407: What age classes were sampled in these other studies?

Thank you yes, I added this to the methods section. The Castillo-Olguin and colaborators (2009) study sampled juveniles (60-130cm long).Nance et al. (2011) paper were juveniles 1-3 years old except adults in Manta Island (TL ≥ 1.5m). Quintanilla et al. (2015) juveniles 30-50 TL except Malpelo Island (adults TL ≥ 1.5m).

36. Line 415: The discussion on relatedness could be built upon more. For example, what were the challenge for assessing sibship in this study? Could take some of the analysis further as well to support more discussion, as mentioned in previous comments.

Yes, I took some of these analyses further.

37. Lines 423-429: This ought to be discussed in the population structure section. Philopatry is the logical explanation for the observed population structure, so integrate there. I also suggest either doing additional analyses on relatedness to develop this section more fully, or use the relatedness statistics to support the population structure findings.

I understand that this paragraph relates to the population structure section. Philopatry in this case is generating the population structure observed, so I still consider that is should be in the section of Relatedness and Natal Philopatry.

38. Figure 1: Add sample sizes to caption or figure

Added

39. Figure 2: It is difficult to see the ticks or count them.

Yes, I added some lines for them to be more visible

40. Figure 5: What is the difference between the gray and black arrows? Specify in the caption.

Ready, the darker the arrows the higher the relative gene flow.

**Reviewer #3.**

Thank you so much for your valuable comments, here I address each of them:

1. Lines 50-51: I am not sure what the value of reporting Ho and allelic richness at microsatellite markers in the abstract is. These are very highly dependent on marker type (bialellic, tri-alellic, how where the markers selected).

You are right, this is not a very substantial result, I will eliminate it from the abstract.

2. Line 87: i would rephrase as "allele frequency differences through time", since divergence usually refers to accumulation of mutations, while here the authors are talking of the effects of drift.

I agree. I will rephrase it with the word differentiation instead of divergence.

3. Lines 214_216 and in general STRUCTURE analyses:The authors do not explain how they chose the value for K they report in the results. What method was used to choose K (Evanno’s method? Other)?

Yes, Evanno’s method was used to choose K

4. How do STRUCTURE plots look for different values of K? IS there any way to assess the admixture proportions? For SNPs data it's common to use evalAdmix (<http://www.popgen.dk/software/index.php/EvalAdmix> ), to evaluate pairwise correlation of residuals matrix between individuals. I am not sure whether there is an equivalent approach for microsatellite data.
   Also, the admixture proportions reported in the figure are a bit difficult to reconcile with both the general population structure (Fst) and relative migration rates inferred: how is it that PAN and GUA show the lowest relative migration rates but the highest levels of admixture?

I re-ran the STRUCTURE analyses and present the plots for different values of K as you suggested. Levels of admixture now coincide with other population structure analyses.

5. I am also not sure why the Authors have not reported structure analyses and DAPC of the entire dataset (including the samples from previous studies).

Structure and DAPC analyses were done only for the locations where I had microsatellite loci genotype data. This was only for the locations of Costa Rica: Ojochal (OJO) and Cocos Island (ICO); Guatemala (GUA) and Panamá (PAN)

6. DivMigrate analyses: what measure of genetic differentiation was used to estimate relative migration patterns?

Dest was used to estimate relative migration patterns

7. Also please give more details on the method (including citation of the method implemented in divMigrate: Sunqvist et al 2016, Ecol Evol <https://doi.org/10.1002/ece3.2096> ). How are relative migration rates scaled? i.e. is the highest migration rate given as 1? An important concern is that this method assumes migration-drift equilibrium, I doubt this is a reasonable assumptions when it comes to long-lived marine animals with large Ne whose habitat has been affected by glaciations. See for example Maisano-Delser et al paper in Heredity on black-tip reef sharks and Walsh et al. paper in Heredity on grey reef sharks. So these results need to be interpreted with caution (as the authors of the package diveRsity themselves say).

Yes, thank you I provide more details on this method. The “divMigrate” function was used to plot the relative migration levels and detect asymmetries in gene flow patterns, between pairs of population samples using DEST values of genetic differentiation (Sundqvist et al., 2016). This function plots sampling areas connected to every other by two connections that represent the two reciprocal gene flow components between any pair of locations (Sundqvist et al., 2016). This approach provides information on the direction of migration using relative migration scales (from 0 to 1) in which the highest migration rate given is 1 (Sundqvist et al., 2016).

Yes, these articles you mention make a thorough analysis of past and present demographics of these species. They have more statistical power, since they are using a large genomic data set of SNPs. Even with this statistical power, these authors mention how equilibrium models of population structure are not realistic and can give misleading results. They mention how increases in Ne can be confounded by increases in migration. They mention how population bottlenecks can also be mistaken for what really is a low number of migrants associated to a metapopulation structure. The genetic variability produces a similar pattern. Thank you for mentioning them to me, but I consider this analyses are a way to visualize other population structure analyses like DAPC, genetic differentiation indexes and STRUCTURE analyses. This graphs provide an idea of how much variation is shared between locations. Also knowing that sampling sizes are different, for example between Cocos Island (N = 15) and areas of the coast (N = 50) there is a big difference of sample sizes, this could be affecting the way connectivity is observed, being highest from the island to the coast, and lowest from the coast to the island. Still I mention in the results that the asymmetry of gene flow is not significant.

8. The authors mention low levels of genetic diversity (referring to pi and haplotype diversity). Low with respect to what? Other populations of the same species, other coastal sharks, or other marine fish? a pi of 0.0016 does not seem very low, but again this depends on what the reference is. What does seem interesting is the high degree of geographical heterogeneity in these estimates.

I re-wrote this section of the discussion. These values can be compared to other related species of sharks from the ETP, like Spyrna zygaena that has Haplotype diversity of 0.86 and nucleotide diversity of 0.26 (Feliz-Lopez., 2019). This species in the south pacific h=0.615 (Hernández., 2013). *Sphyrna tiburo* h = 0.932 Escatel-Luna., 2015. And *S. lewini* in the ETP with previous studies h=0.53 Nance et al., 2011 and Castillo-Olguin h=0.49.

9. Regarding migration rates, the authors mention that “Analysis of the extent and direction of gene flow showed no significant movement between coastal sampling sites.”. I am not sure how this conclusion was reached. There is no real analyses of the extent of gene flow, as measures of geneflow are "relative" (no absolute values ). Also the analyses assume migration-drift equilibrium and an island model, so the authors must be careful in interpreting the results.

Yes, this was written in a confusing way. What this analysis did was test if there was asymmetric gene flow between these areas, which was not found. The statement should be corrected the following way:

Analysis of the extent and direction of gene flow showed no significant asymmetric movement between coastal sampling sites.

10. The authors mention that the low diversity of mtDNA is consistent with overexploitation. (Lines 352-353). No evidence is presented that the low levels of genetic diversity of this species are linked in any way to recent population declines. Given the generation time of scalloped hammerheads i find this hypothesis extremely unlikely.
    None of the analyses the author presented allow any inference of recent changes in Ne, and to my knowledge such analyses would require extensive two-locus statistics (LD) obtained for a great portion of the genome, along with good linkage maps (for example, using the method developed by Santiago: <https://doi.org/10.1093/molbev/msaa169>) . Also please note that most studies on genetic diversity of sharks concluded that patterns of genetic diversity were almost certainly unrelated to recent population declines but rather reflect the species history of colonization/range expansion/isolation. See work on grey nurse sharks (Stow et al 2006 Biology Letters and subsequent paper in Molecular Ecology about grey nurse sharks <https://doi.org/10.1098/rsbl.2006.0441> <https://doi.org/10.1111/j.1365-294X.2009.04377.x> , and recent work on blacktip reef shark by Stefano Mona and Maisano-Delser <https://doi.org/10.1038/s41437-018-0164-> , as well as work on grey reef sharks just published in heredity <https://doi.org/10.1038/s41437-022-00514-4> ).
    If the authors want to test this hypothesis they could try to use the R package “migraine” to detect possible bottlenecks, but they should also be aware that these estimates could be biased by complex demographic histories (e.g. <https://doi.org/10.1038/s41437-018-0164-0> ).

Thank you, I addressed these observations in the first paragraph of the Genetic diversity part of the Discussion

11. Lines 368-369: I am not sure what is meant by "non-detectable bottleneck effect". It could very well be that overharvesting may have reduced census size while having negligibly effects on effective population size. A non-detectable effect is not an effect at all?

Yes, I realize this is not clear. When I did the bottleneck analyses, there was none detected. So yes, it is not an effect. After consideration, I think that these results of the bottleneck analysis are not relevant. Other reviewers also suggested to eliminate this since it does not contribute much.

Changes in the reference list:

In the introduction this is a very important article that gives context of elasmobranch conservation in Costa Rica

ADDED: Espinoza M, Díaz E, Angulo A, Hernández S, Clarke TM. Chondrichthyan diversity, conservation status, and management challenges in Costa Rica. Frontiers in Marine Science. 2018.

These two programs were used to make the figures that include maps, I had not cited them previously

ADDED: ESRI. ArcMap Version 10. Redlands California; 2020.

ADDED: QGIS Development Team. QGIS Geographic Information System.Open Source Geospatial Foundation Project. 2020.

I had not cited the AMOVA method properly before:

ADDED: Excoffier L, Smouse PE, Quattro JM. Analysis of molecular variance inferred from metric distances among DNA haplotypes: Application to human mitochondrial DNA restriction data. Genetics. 1992;131(2):479–91.

I eliminated the analyses of relatedness using this program, so I no longer cite it.

REMOVED: Kalinowski ST, Wagner AP, Taper ML. ML-RELATE: A computer program for maximum likelihood estimation of relatedness and relationship. Mol Ecol Notes. 2006;

For genetic differentiation indexes, and the obtainment of confidence intervals I used the DiveRsity R package instead of the DEMEtics R package.

ADDED: Keenan K. DiveRsity: A comprehensive, general purpose population genetics analysis package. R package version 1.2017

REMOVED: Jueterbock A, Kraemer P, Gerlach G, Deppermann J. DEMEtics: evaluating the genetic differentiation between populations based on Gst and D values. Mol Ecol. 2011;

I added these references for population structure analyses

ADDED: Pritchard JK, Stephens M, Donnelly P. Inference of population structure using multilocus genotype data. Genetics. 2000;155(2):945–59.

ADDED: Evanno G, Regnaut S, Goudet J. Detecting the number of clusters of individuals using the software STRUCTURE: A simulation study. Mol Ecol. 2005;14(8):2611–20.

For the paragraph on genetic diversity I eliminated this reference:

REMOVED: Hadi S, Andayani N, Muttaqin E, Simeon BM, Ichsan M, Subhan B, et al. Genetic connectivity of the scalloped hammerhead shark Sphyrna lewini across Indonesia and the Western Indian ocean.PLoS One. 2020

and added 6 new references

ADDED: Green ME, Appleyard SA, White WT, Tracey SR, Heupel MR, Ovenden JR. Updated connectivity assessment for the scalloped hammerhead (Sphyrna lewini) in Pacific and Indian Oceans using a multi-marker genetic approach. Fish Res [Internet]. 2022;251(November 2021):106305. Available from: https://doi.org/10.1016/j.fishres.2022.106305

ADDED: Escatel-Luna E, Adams DH, Uribe-Alcocer M, Islas-Villanueva V, Díaz-Jaimes P. Population genetic structure of the Bonnethead Shark, sphyrna tiburo, from the western north Atlantic Ocean based on mtDNA sequences. J Hered. 2015;

ADDED: Félix-López DG, Bolaño-Martinez N, Díaz-Jaimes P, Oñate-González EC, Ramírez-Pérez JS, García-Rodríguez E, et al. Possible female philopatry of the smooth hammerhead shark Sphyrna zygaena revealed by genetic structure patterns. J Fish Biol. 2019;94(4):671–9.

ADDED: Bolaño-Martínez N, Hernández-Muñoz S, Uribe-Alcocer M, Galván-Magaña F, Ritchie PA, García-De León FJ, et al. Population genetic divergence as consequence of past range expansion of the smooth hammerhead shark Sphyrna zygaena. Hydrobiologia. 2019;837(1):31–46.

ADDED: Feutry P, Kyne PM, Pillans RD, Chen X, Naylor GJP, Grewe PM. Mitogenomics of the Speartooth Shark challenges ten years of control region sequencing. BMC Evol Biol. 2014;14(1):1–9.

ADDED: Martin AP, Naylor GJP, Palumbi SR. Rates of mitochondrial DNA evolution in sharks are slow compared with mammals. Nature. 1992;357(6374):153–5.
