# Supplementary material for: Population structure and genetic connectivity of the scalloped hammerhead shark (Sphyrna lewini) across nursery grounds from the Eastern Tropical Pacific: Implications for management and conservation
Source: PLoS One. 2022 Dec 16;17(12):e0264879. doi: 10.1371/journal.pone.0264879 (PMC9757582; doi:10.1371/journal.pone.0264879)
Supplement: Attachment — Submitted filename: Response to reviewers 17.08.2022.docx [file pone.0264879.s013.docx]

Response to Reviewers

6. Review Comments to the Author

Reviewer #3: The authors addressed most of my concerns.


A further comment to be addressed in a minor revision (no need to send this back to me for review)


In the discussion about genetic diversity, the authors mention the slow mutation rate for mtDNA in sharks. Note that genetic diversity is a product of Ne and mutation rate, i.e. nucleotide diversity is determined by the mutation scaled population size (or the population scaled mutation rate, if you prefer). So that at equilibrium pi= = 4Ne (where is the mutation rate). So the authors are correct that the mutation rate alone does not explain low nucleotide diversity, but this does not suggest at all that nucleotide diversity could reflect overexploitation. It most likely (given generation time and time for pi to reach equilibrium) reflects historical demographic events. So the point that diversity is likely shaped by long term Ne or other demographic events should be made, in my opinion. Indeed, theta (and pi at equilibrium) are indeed a measure of population size given a mutation rate

Thank you, I addressed all the suggestions of low nucleotide diversity reflecting overexploitation in the Manuscript. In order to do so, I eliminated the following citations from the reference list:

78. Chapman DD, Pinhal D, Shivji MS. Tracking the fin trade: Genetic stock identification in western Atlantic scalloped hammerhead sharks Sphyrna lewini. Endanger Species Res. 2009;

79. Baum JK, Myers RA, Kehler DG, Worm B, Harley SJ, Doherty PA. Collapse and conservation of shark populations in the Northwest Atlantic. Science (80- ). 2003;
